# Meta‐barcoding insights into the spatial and temporal dietary patterns of the threatened Asian Great Bustard (*Otis tarda dybowskii*) with potential implications for diverging migratory strategies

**DOI:** 10.1002/ece3.3791

**Published:** 2018-01-08

**Authors:** Gang Liu, Aaron B. A. Shafer, Xiaolong Hu, Linhai Li, Yu Ning, Minghao Gong, Lijuan Cui, Huixin Li, Defu Hu, Lei Qi, Hengjiu Tian, Bojun Wang

**Affiliations:** ^1^ Research Institute of Wetland Beijing Key Laboratory of Wetland Services and Restoration Chinese Academy of Forestry Beijing China; ^2^ Forensics & Environmental and Life Sciences Trent University Peterborough ON Canada; ^3^ College of Nature Conservation Beijing Forestry University Beijing China; ^4^ State Forestry Planning and Design Institute of Forest Products Industry Beijing China; ^5^ Beijing Wildlife Rescue and Rehabilitation Beijing China

**Keywords:** Great Bustard, molecular diet analysis, partial migration, spatiotemporal changes, wintering food

## Abstract

Food resources are often not sufficient to satisfy the nutritional and energetic requirements during winter conditions at high latitudes. Dietary analysis is a prerequisite to fully understanding the feeding ecology of a species and the nature of trophic interactions. Previous dietary studies of Asian Great Bustard (*Otis tarda dybowskii*) relied on behavioral observations, resulting in categorization of diet limited to broad taxonomic groupings. Here, we applied a high‐throughput sequencing meta‐barcoding approach to quantify the diet of resident and migratory Asian Great Bustard in three wintering sites during early winter and late winter. We detected 57 unique plant taxa in the bustard diet, among which 15 species were confirmed by a local plant database we generated. Both agricultural and natural foods were detected, indicating a relatively broad dietary niche. Spatiotemporal dietary changes were discovered, revealing diet differences among wintering sites and a general shift toward lower plant diversity later in winter. For the nonmigratory population, we detected a significantly more diverse array of plant species in their diet. We hypothesize that dietary variation between resident and migratory populations could be involved in the recent transition to partial migration in this species, although climate change can not be excluded. Collectively, these results support protecting unharvested grain fields and naturally unplowed lands to help conserve and promote population growth of Asian Great Bustard.

## 1 INTRODUCTION

Land‐use alteration and habitat loss are increasing across the globe (Sala & Wall, 2000), with the direct consequences of altering the distribution of feeding sites and the availability of food resources (Martinson & Fagan, 2014; Pezzanite, Rockwell, Davies, Loonen, & Seguin, 2005). A decrease in forage quantity and quality can affect individual growth rates, phenotypic variability, fecundity, predation, population abundance, and distribution (Piersma & Drent, 2003; Taillon, Sauvé, & Côté, 2006). These negative effects can be exaggerated under harsh winter conditions at high latitudes where food resources might not be sufficient to satisfy the nutritional and energetic requirements of the organism (Bartoń & Zalewski, 2007; Forsman & Mönkkönen, 2003; Johnsen et al., 2017). Importantly, nutrient‐limited winters have been shown to alter individual behavior and delay breeding, ultimately having negative fitness consequences (Danner, Greenberg, Danner, & Walters, 2014; Gordo, Brotons, Ferrer, & Comas, 2005).

The importance of food limitation has been extensively studied in terrestrial vertebrate species by experimentally manipulating food supply (Cooper, Sherry, & Marra, 2015; Ruffino, Salo, Koivisto, Banks, & Korpimäki, 2014); however, diet compositions and requirements are unknown for most wild populations. Diet analysis plays a crucial role in the feeding ecology and trophic interactions of a species (Montoya, Pimm, & Solé, 2006) and is vital information for species conservation and management, particularly those in captivity or under supplemental feeding regimes (Jordan, 2006). For most natural populations, it is near impossible to accurately and efficiently characterize the complex composition of a diet. This is because traditional diet analyses, that include direct field‐collected observations, microhistological identification of stomach or fecal content, near‐infrared reflectance spectroscopy, stable isotope analysis, cafeteria experiments in artificial environments, and combinations of the above require extensive resources and expertise with inherent limitations (Piñol, San Andrés, Clare, Mir, & Symondson, 2014; Pompanon et al., 2012; Valentini, Pompanon, & Taberlet, 2009). By comparison, the application of high‐throughput sequencing (HTS) and the effective combination of DNA meta‐barcoding has enabled dietary analysis of field‐collected feces, thereby providing detailed inventories of diet in wild organisms (Deagle, Thomas, Shaffer, Trites, & Jarman, 2013).

In many migratory bird species, increased mortality is often seen during stationary periods in winter, especially for birds relying on farmlands (Klaassen et al., 2014). The Great Bustard (*Otis tarda*) is a globally threatened migratory species that formerly occupied steppe regions and lowland grasslands across Eurasia. There are two recognized subspecies, nominal (*O. t. tarda*) and Asian (*O. t. dybowskii*), geographically separated by the Altai Mountains in east‐central Asia (Kessler & Smith, 2014). The subspecies differ significantly in population size with the nominal subspecies estimated at 50,000 and the Asian subspecies at around 1,500–2,200 (Alonso & Palacín, 2010; Kessler, 2015). The overall population of the nominal Great Bustard is considered stable, but the population size of Asian Great Bustard has been declining due to agricultural intensification, habitat degradation, and illegal hunting (Alonso & Palacín, 2010).

Detailed diet studies have previously been undertaken on *O. t. tarda* (Alonso, Alonso, & Naveso, 1999; Bravo, Ponce, Bautista, & Alonso, 2016; Bravo, Ponce, Palacín, & Alonso, 2012; Lane, Alonso, Alonso, & Naveso, 1999; Rocha, Marques, & Moreira, 2005), but studies on the diet of Asian Great Bustard have relied only on behavioral observation (Li et al., 2005) resulting in a superficial knowledge of diet with information limited to broad taxonomic designations. With the feeding quantity and quality in winter severely reduced, food shortages have regularly occurred in some wintering grounds in China (Wang, 2012; Wu, Shen, et al., 2013) and modern agricultural fields have little food to offer wintering bustards, which might influence the nutritional status and diet selection of individuals at their wintering ground (Wu, Liu, Xu, & Liu, 2013). Additionally, land‐use practices in China and climate change are increasing the extent of the Gobi Desert, a major migratory obstacle with limited forage for migrating bustards (Mi, Falk, & Guo, 2016; Wang, Jin, & Nimmo, 2008).

The Asian Great Bustard is no longer observed to overwinter in previous wintering grounds in the Yangtze region, and the wintering range appears to have contracted northward to the Yellow River (Liu et al., 2017). Interestingly, evidence of partial migration, meaning some individuals have stopped migrating, has recently been reported (Li et al., 2005; Yu, Qiao, Zou, Yang, & Sun, 2008). For example, in the Tumuji Nature Reserve, a few individuals of Great Bustard were observed overwintering in the 1990s, and this has persisted for the last decade (Figure S1). In birds, partial migration is a relatively common phenomenon, particularly in areas with seasonal fluctuation of food availability and severe climate (Chapman, Brönmark, Nilsson, & Hansson, 2011; Olsson, Greenberg, Bergman, & Wysujack, 2006; von Rönn, Shafer, & Wolf, 2016). Partial migration in birds has been hypothesized to be driven by dominance (Smith & Nilsson, 1987), arrival time (Lundberg, 1988), and body size (Belthoff & Gauthreaux, 1991); all hypotheses assume that food scarcity or physiological intolerance to climatic conditions (or both) limit the ability of individuals to remain on their breeding grounds during the nonbreeding season (Boyle, 2008). As partial migration of birds has a hypothesized link to variation in food abundance, quantifying the spatial and temporal variations in food resources might explain why some individuals choose to remain year‐round while others migrate.

The main aim of this study was to characterize the diet of wintering Great Bustards in early winter and late winter by meta‐barcoding of noninvasively collected fecal samples. We tested the predictions that (1) there is spatial variance in the diet of wintering Great Bustards and (2) plant diet diversity decreases as the winter progresses. The findings are discussed in light of the conservation status of this enigmatic species and implications for our understanding of the evolution of partial migration.

## 2 MATERIALS AND METHODS

### 2.1 Study area

**Figure 1:**
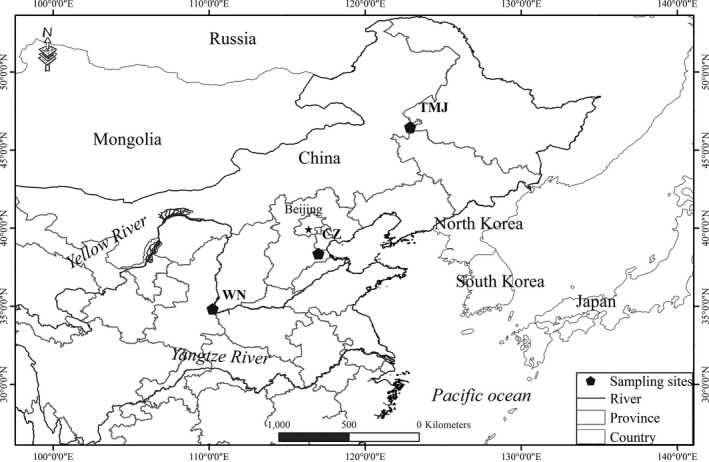
Fecal sampling locations of Asian Great Bustard in China. The sampling site includes Tumuji Nature Reserve (TMJ) in inner Mongolia Autonomous Region, Cangzhou (CZ) in Hebei Province, and Weinan (WN) in Shaanxi Province

The Tumuji Nature (TMJ) Reserve lies in the transition zone between temperate steppe and meadow steppe in northeastern Inner Mongolia, and covers approximately 76,210 ha (Figure 1). Farmland makes up an additional 30,280 ha of the reserve, with corn (*Zea mays*) and mung bean (*Vigna radiata*) fields as dominant agricultural fields, and soybean (*Glycine ma*x) and sorghum (*Sorghum bicolor*) also being cultivated. The TMJ Reserve is the most important breeding site for Asian Great Bustard in China, but part of the breeding population also overwinters in the TMJ Reserve (Figure S1). Cangzhou (CZ) is located in the western coastal plain of Bohai Bay and is an important stopover site and wintering ground; approximately 200 Asian Great Bustard per year overwinter in CZ (Meng, 2010; Wu, Hou, & Gao, 2010). The dominant agricultural fields are corn and winter wheat (*Triticum aestivum*). The Weinan (WN) area is another important wintering ground for the Asian Great Bustard and consists largely of agricultural fields near the confluence of the Wei and Yellow Rivers in Shaanxi Province of China (Kessler, Batbayar, Natsagdorj, Batsuur, & Smith, 2013). The WN wintering ground is approximately 60,000 ha with winter wheat and corn as dominant agricultural fields, and more than 200 individuals have been observed wintering here (ca. 2015; data unpublished).

### 2.2 Fecal sampling and dietary analysis

Fecal sampling was conducted in TMJ, CZ, and WN in early winter (November 2015) and late winter (February 2016). Fresh fecal samples were collected throughout the three study areas, at places where Asian Great Bustard were observed, primarily near roosting sites. When birds had flown away for natural reasons, the feces were randomly sampled in each wintering roost. To minimize the probability of recollecting fecal samples from the same individual, a wintering roost study area was monitored only once and sample collection was randomly conducted from as many roosts as we could observe in each study area (see Liu et al., 2017 for additional information on sampling protocol). Samples collected in TMJ were from resident Great Bustards, and samples in CZ and WN were from migratory individuals (Figure 1). All fecal samples were stored in a cooler during sample collection, then preserved at −20°C. DNA from fecal samples was extracted using the QIAamp Fast DNA Stool Mini Kit (QIAGEN, Germany). DNA extractions were performed in a clean room, and two negative controls were included to monitor contamination.

Many studies indicate that plant items are the main food resources for Great Bustard in winter (Bravo et al., 2012; Gooch, Ashbrook, Taylor, & Székely, 2015), and vertebrate and invertebrate remains in diet are not our focus in this study. The universal P6 loop of the chloroplast *trn*L (UAA) intron was amplified by PCR (Taberlet et al., 2007). The 15 μl PCR consisted of 1× Biomed Taq PCR MasterMix, 0.1 μmol/L of each primer, 2.5 μl DNA extract, and 0.1 μl bovine serum albumin (10 mg/ml, Takara, Japan). The PCR mixtures were denatured at 95°C for 10 min, followed by 50 cycles at 94°C for 30 s, 54.6°C for 60 s, and 72°C for 10 min. The primers were modified to include barcodes using Oligotag (Coissac, 2012). The primers were as follows, with x denoting the barcode: G, x ‐5′‐GGGCAATCCTGAGCCAA‐3′, and H, x‐5′‐CCATTGAGTCTCTGCACCTATC‐3′. Barcodes are included in Table S1. The PCR products were purified using the QIAquick PCR Purification Kit (QIAGEN, Germany) and pooled in equal volume. Purified amplicons were then ligated to Illumina adaptors, and HTS was carried out on MiSeq 2000 150PE (Illumina Inc., USA).

### 2.3 Construction of reference databases from potential dietary species

Reference databases of chloroplast *trnL* intron of plants were assembled using the ecoPCR program (Ficetola et al., 2010) and sequences derived from public databases (National Center for Biotechnology Information, NCBI; European Molecular Biology Laboratory, EMBL; DNA Data Bank of Japan, DDBJ) and sequences of local plants. For the latter, a local database was established by collecting leaves of plant species available to the Great Bustard in the field. After being identified to the species level morphologically, leaf tissues were stored in envelopes with silica gel. Plant DNA was extracted using the Rapid Extraction Kit (Biomed, China) and ground under liquid nitrogen. Fragments of the chloroplast “barcoding” regions were amplified representing *trnL*‐F (615‐955 bp) using the primer pairs trnL c and f (Taberlet, Gielly, Pautou, & Bouvet, 1991). PCR was performed on 2.5 μl of DNA extract in a 25 μl volume containing 2× Taq PCR MasterMix (Biomed), 0.2 μmol/L of each primer. Thermo cycling conditions were as follows: 95°C for 5 min, 30 cycles of 94°C for 30s, 56.6°C for 1 min, 72°C for 1 min, and with a final incubation at 72°C for 10 min. Sequencing was performed using BigDye Terminator v3.1, and products were analyzed in both directions on an ABI 3100 Genetic Analyzer (Sangon, Shanghai, China).

### 2.4 Sequence analysis and filtering

The analyses of raw sequence reads were carried out using OBITools (Boyer et al., 2016). The script *illuminapairedend* was used to assemble direct and reverse reads to a single sequence, and primers and tags were demultiplexed and filtered using the *ngsfilter* script as described in Shehzad et al. (2012b). Sequences shorter than 10 bp and longer than 150 bp, or with sequence counts lower than 1,000 were excluded. The *obiclean* script was then run to assign each sequence within a PCR product the status of “head,” “internal,” or “singleton” (Shehzad et al., 2012a), according to a directed acyclic graph, as described in De Barba et al. (2014). Sequences not identified by the *obiclean* script as “head” in ≥3 samples or “singleton” in four samples were considered erroneous and removed. Taxon assignment was then achieved using the *ecotag* program to find highly similar sequences against the reference databases. Taxonomically assigned sequences having a relatively low frequency of occurrence underwent further filtering and were discarded if the frequency of occurrence (number of fecal samples) for a given sequence was below a 5% threshold.

To increase accuracy of the automatic taxonomic assignation and exclude chimeric sequences, we considered only sequences with a specified identity threshold of ≥95% over the entire query sequence length with any reference sequence and refined it with the known distribution of Asian Great Bustard food resources in the study area. Sequences were grouped into molecular operational taxonomic units (MOTUs) using the following sequencing similarity threshold: Sequences with identity ≥99% to a single species within the local reference library or the public library were considered as a “species match,” and as a “genus match” if sequences had ≥98% similarity to one or more species within the same genus (Burgar et al., 2014).

### 2.5 Diet analysis

Multivariate analyses of the plant diet data were performed, with sample categories grouped by wintering site (TMJ, WN, and CZ) and wintering time (early winter and late winter). The proportions of plant families in the diet composition of each category were plotted using the sum of sequence reads. We used a species rarefaction curve to estimate the total number of plant species likely to be eaten by each group. The species accumulation, based on the fecal samples, was computed in ESTIMATES Version 8.2 (Colwell & Elsensohn, 2014), with a randomization step of individuals that was repeated 100 times.

Dietary differences were compared using two standardized indices: (1) mean fecal occurrence frequency (proportion of the samples containing a given plant species) and (2) mean plant taxa per fecal sample (mean number of plant species in a given sample). Alpha diversity indices (Simpson index and Chao1 index) were computed to assess dietary diversity for each group at the MOTU level using species number and read counts data. The values of Simpson and Chao1 were calculated in R 3.4.1 (R Development Core Team 2017). A Box‐Cox transformation was applied to abundance data to improve conformity to normality assumptions prior to Simpson diversity analysis. Differences in alpha diversity were compared by wintering site and by wintering time. The one‐sample Kolmogorov–Smirnov (K–S) test was calculated to test the normality of the data with an independent‐sample *t* test (for the normally distributed data) or Mann–Whitney *U*‐test (for the non‐normally distributed data) applied to compare diversity changes between early winter and late winter with the same wintering site. A one‐way ANOVA (for the normally distributed data) or Kruskal–Wallis test (for the non‐normally distributed data) was used to examine the effect of wintering site on diversity. Tukey's HSD post hoc tests were conducted to detect the homogeneity of group variances.

To evaluate the pattern of dispersion of samples within each group, beta diversity was calculated with the Bray–Curtis distance matrices (Beals, 1984) using 9999 permutations in the R Vegan package. Diversity was compared among wintering sites and between sampling times to assess spatiotemporal differences in diet composition. The interactive effect between site and time was also examined, and Tukey's HSD post hoc tests were run to determine the variance between groups. Nonmetric multidimensional scaling (NMDS) based on the Bray–Curtis distance matrix was applied to visualize spatial and temporal variations in diet between each group. A threshold of stress value of <0.2 was used for the NMDS plot. To validate the NMDS, principal coordinate analysis (PCoA) plots were generated in R 3.4.1. Tests for significant differences in diet composition between wintering sites and wintering time were performed by nonparametric Analysis of Similarity (ANOSIM) in R 3.4.1. For each wintering time (early winter and late winter), a Similarity Percentages analysis (SIMPER) (Clarke, 1993) using PRIMER v6 (Clarke & Gorley, 2006) was performed to determine which plant species were responsible for the dissimilarity among wintering sites.

## 3 RESULTS

In the two sampling expeditions (in early winter and late winter), we collected 15 and 16 fecal samples from TMJ, 16 and 16 from WN, and 16 and 17 from CZ. We could not reliably sex the feces as the Asian Great Bustard was observed in mixed flocks in our study area. In addition to collating sequences from public repositories, we sequenced the P6 loop of the chloroplast *trnL* (UAA) intron in 99 plant specimens, resulting in 78 plant species distributed across 29 families and 70 genera (Table S2). The length of sequences ranges from 71 to 160 bp. These sequences were added to the reference databases constructed from NCBI, EMBL, and DDBJ.

High‐throughput sequencing of all samples generated a total of 38.5 million paired‐end sequence reads (Table 1), which were separately processed and filtered for different wintering sites (Table 1). A total of 4.3 million reads were retained for the whole diet data set after the sequence filtering process (Table 1). The rarefaction curves showed that sequencing depth was sufficient to capture the entire plant species diversity in feces at each site over each wintering period (Figure S2).

**Table 1:** Summary of the number of sequences and samples after different steps of the data filtering protocol for the fecal samples analyzed. The proportion of sequences remaining from the previous filtering step is indicated in parentheses. TMJ, CZ, and WN represent Tumuji Nature Reserve, Cangzhou, and Weinan, respectively

Processing steps	Wintering sites		
	TMJ	WN	CZ
Estimated paired‐end sequences reads (%)	14,136,271	11,982,468	12,385,493
Sequence reads for which primers and tags were identified (%)	4,756,617 (33.6)	2,755,103 (22.9)	3,011,259 (24.3)
Unique sequences (%)	174,698 (3.7)	217,619 (7.8)	136,781 (4.5)
Unique sequences >10 bp and <150 bp (%)	152,422 (87.2)	164,975 (75.8)	114,370 (83.6)
Unique sequences >10 bp and <150 bp, with counts >1000 (%)	319 (0.2)	303 (0.2)	316 (0.3)
Unique sequences after *obiclean* filtering (%)	94 (29.5)	89 (29.4)	94 (29.7)
Unique sequences with best identity ≥95% (%)	32 (73.3)	23(77.4)	21 (64.7)

### 3.1 Diet of wintering Asian Great Bustard

**Figure 2:**
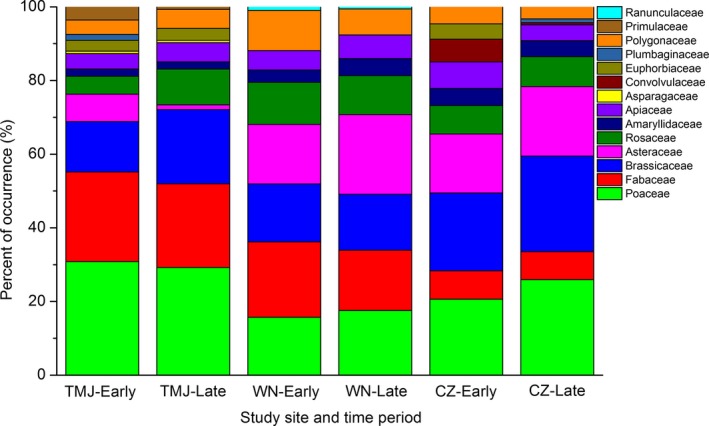
Percent of occurrence of plant taxa at the family level identified in the Asian Great Bustard fecal samples. TMJ, CZ, and WN represent Tumuji Nature Reserve, Cangzhou, and Weinan, respectively

In the 96 fecal samples analyzed, 57 MOTUs were detected after filtering sequences based on reference databases, with between 3 and 28 MOTUs per fecal sample. A total of 20 MOTUs could be identified to species, among which 15 species were confirmed by screening the local reference database (Table 2). In early winter, the number of MOTUs was 32 for TMJ, 19 for CZ, and 22 for WN (Table 2), while in late winter, the MOTUs for TMJ, WN, and CZ were 27, 22, and 20, respectively. Overall, winter diet items consisted of 14 families of plant. Fabaceae, Brassicaceae, Asteraceae, and Poaceae accounted for more than 70% reads reflecting the diet (Figure 2, Figure S3). The dominant species in terms of both frequency of occurrence and numerical abundance were *Vigna radiata*,* Zea mays*,* Potentilla discolor*,* Descurainia sophia,* and *Oxytropis racemosa* for the partially migratory TMJ population, *Glycine max*,* Potentilla discolor*,* Rumex spp*., and *Adenocaulon bicolor* for the migratory WN population, and *Zea mays*,* Triticum aestivum*, and *Descurainia sophia* for the migratory CZ population (Table S3).

**Table 2:** Number of plant taxa identified to each categorized class in diet of Asian Great Bustard. TMJ, CZ, and WN represent Tumuji Nature Reserve, Cangzhou, and Weinan, respectively

Wintering site	Wintering period	Number of taxons	Identified to family	Identified to genus	Identified to species	Identified to local database
TMJ	Early	32	12	7	13	11
	Late	27	11	5	11	11
WN	Early	22	5	6	10	5
	Late	22	6	5	10	5
CZ	Early	19	8	2	9	5
	Late	20	8	3	9	5

### 3.2 Spatiotemporal variation in diet

**Figure 3:**
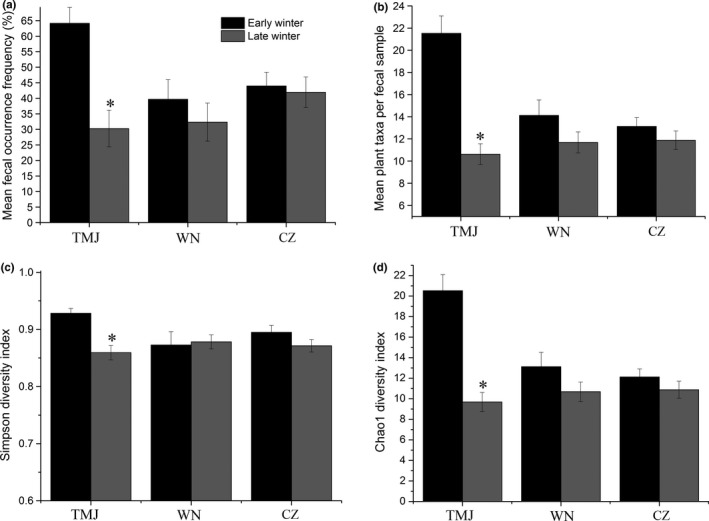
Diet changes of the Asian Great Bustard during early winter and late winter and dietary diversity indices. (a) Mean fecal occurrence frequency (%); (b) mean plant taxa per fecal sample; (c) Simpson diversity index; and (d) Chao1 diversity index. Statistical differences are indicated by * (*p* < .05). Error bars are the standard error. TMJ, CZ, and WN represent Tumuji Nature Reserve, Cangzhou, and Weinan, respectively

The mean frequency of occurrence, meaning the proportion of samples containing a given prey taxa, differed in TMJ between early winter (64.16 ± 5.11%) and late winter (30.27 ± 5.89%) (*Z* = −4.06, *p* < .01), while no significant temporal changes were found in the migratory populations (WN and CZ) (Figure 3a). Mean number of plant taxa per fecal sample for resident Great Bustards was reduced in late winter for TMJ (*Z* = −4.04, *p* < .01) (Figure 3b), but not for WN and CZ. The percent occurrence of weeds was higher than cultivated plants for each sampling population, and this decreased as the winter progressed; the percent of occurrence of cultivated plants remained stable throughout the winter (Figure S4).

Both alpha diversity indices indicated spatial differences in dietary diversity among wintering populations in early winter and late winter (Figure 3c, Table S4), except for the Chao1 index among populations in late winter (Figure 3d). Temporally, there were differences in the Simpson diversity levels in TMJ (*t* = 4.45, *p* < .01; Figure 3c). For the Chao1 diversity index, there were temporal differences in TMJ, with a value of 20.5 in early winter and 9.7 in late winter (*Z* = −4.04, *p* < .01; Figure 3d). Post hoc tests of the Simpson index indicated Great Bustards in TMJ had higher dietary plant diversity than WN and CZ populations in early winter, whereas Great Bustards in TMJ showed lower diversity than WN and CZ populations in late winter (Table S4). Analysis of beta diversity revealed an interactive effect of wintering site and wintering time on diets of Asian Great bustards (*F*_2,90_ = 9.25, *R* ^2^ = .11, *p* < .01; Table S5) and with differences between wintering sites in early winter (*F*_2,44_ = 7.98, *p* < .01), and late winter (*F*_2,46_ = 14.87, *p* < .01). The beta diversity differed between early and later winter in TMJ (*F*_1,29_ = 18.32, *p* < .01), WN (*F*_1,30_ = 6.90, *p* < .01), and CZ (*F*_1,31_ = 6.90, *p* < .01; Table S6).

**Figure 4:**
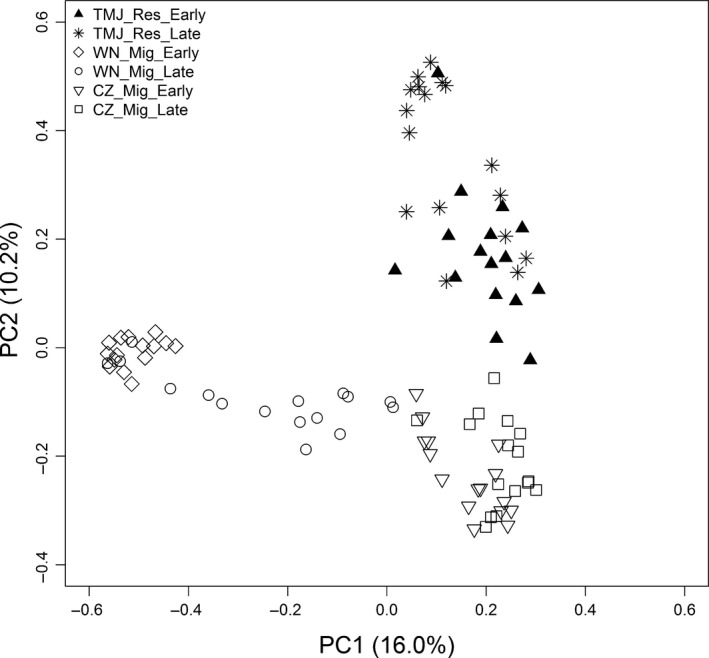
Principal coordinate analysis (PCoA) of molecular operational taxonomic units (MOTUs) for the resident population and two migratory populations of the Asian Great Bustard in early winter and late winter, based on Bray–Curtis distance unweighted by MOTU abundance. Each symbol corresponds to one fecal sample. The first two principal coordinate (PC) axes are shown

The two‐dimensional ordination plot showed the samples sorting relative to their dissimilarity, with similar samples in close proximity and dissimilar samples further apart. The PCoA plot (Figure 4) was consistent with the NMDS plot (Figure S5), revealing a pattern in diet in relation to wintering site and wintering time. Results of the ANOSIM confirmed that diet composition was different among wintering sites and between wintering times (Table S7). Spatial change in diets varied significantly among wintering sites in early winter (*R* = .933, *p* = .001) and in late winter (*R* = .807, *p* = .001). Pairwise comparisons revealed differences between early winter and late winter for all sites (Table S7). The SIMPER analysis indicated that the average dissimilarity among wintering sites was very high (>90%) (Table S8). In early winter, average similarity in diet among sites ranged between 91.4% and 97.6%. In early winter, *Vigna radiata* contributed between 68.1% and 80.9% of the average dissimilarity between sites. Three dominant species (*Glycine max, Potentilla discolor, and Descurainia sophia*) also contributed to this level of dissimilarity (Table S8).

## 4 DISCUSSION

For both resident and migratory populations of wintering Asian Great Bustard, weedy plants and cultivated plants were the main food items detected in feces at the three sampled locations, suggesting a relatively broad dietary niche. This is in general agreement with previous diet studies based on behavioral observations and microhistological identification (Li et al., 2005; Wu, Shen, et al., 2013). For the partially migratory TMJ population, we observed the highest mean frequency of prey items and diet diversity in early winter. This suggests that in early winter, considerably more plant species are available for consumption on the reserve, and most individuals appear to be feeding on the entire variety of resources. We hypothesize this is a contributing factor to the recent shift to nonmigratory strategies in TMJ, as there is a significant difference in species abundance and consumption of plant species between the nonmigratory TMJ birds, and those migrating to WN and CZ. However, we note that climate change and other factors cannot be ruled out (Alonso, Salgado, & Palacín, 2016; Palacín, Alonso, Martín, & Alonso, 2017; Palacín & Martín, 2011).

Cultivated plants appear to play an important role in diets of overwintering Asian Great Bustard, and this is also supported by the studies of wintering habitat selection that Great Bustards often use harvested croplands for foraging and roosting (Lane et al., 1999; Martínez, 2000; Palacín, Alonso, Martín, & Alonso, 2015; Raab, Schütz, Spakovszky, Julius, & Schulze, 2015; Zhao, Yan, Weng, & Zhang, 2011). Changes in cropland use could therefore affect winter seed abundance and vegetation cover, which in turn influence the food supplies of the wintering cropland‐reliant birds such as bustards (Raab et al., 2015; Végvári et al., 2016). For example, herbicide use, crop diversification, the switch from spring‐sown to autumn‐sown, and the shift from one crop to another crop were correlated with lower population levels in farmland birds (Batáry, Matthiesen, & Tscharntke, 2010; Josefsson, Berg, Hiron, Pärt, & Eggers, 2016; Lane, Alonso, & Martin, 2001).

While many bird species have adapted to increases in cropland, the changing weather and climate conditions influence the diversity and quantity of food resources that can make it challenging for organisms to obtain sufficient resources (White, 2008). In winter, the Great Bustards invest most of their daytime foraging, which requires long foraging bouts to gather enough food (Martínez, 2000). Foraging time accounts for as much as 46% of the Asian Great Bustard's behavior budget in Hebei Province, China, during winter (Sun, Li, Li, Wu, & Li, 2006). Interestingly, Asian Great Bustards have been shown to move itinerantly across relatively large winter home ranges (30–95 km) (Kessler et al., 2013), which could be a compensatory strategy to maintain a diverse diet by exploiting a wider variety of feeding habitats. How this varies by sex remains unknown as we could not reliably sex pellets, but this remains an avenue to explore in the future.

Temporal dietary changes across winter were detected in Asian Great Bustard with a general shift toward low dietary diversity in late winter and nonoverlapping resource use at different wintering times. Dietary diversity might be reduced at our study sites due to snow cover, which limits possibilities for foraging; on‐site camera data showed that Asian Great Bustard in TMJ consumed supplemental food (*Vigna radiate*,* Zea mays—*that was detected in our metagenomic assay) after snowfalls. Snow will cover the dormant vegetation and seeds limiting accessibility, and snowfall events have become more common in the range of the Asian Great Bustard (Li, Wang, & Yang, 2013; Wang, He, & Zhang, 2015). Thus, this reflects a case where species develop temporal shifts in diet to adapt to changes of food resource availability (Piersma, Verkuil, & Tulp, 1994; Renton, 2001), and this is particularly evident in winter when food is limited (Renner et al., 2012).

In terms of Great Bustard conservation and management, we suggest reserves maintain unharvested fields (mung bean), stubble fields (recently harvested cereal or legume fields), and naturally unplowed lands for wintering Great Bustard. Farmland provided the main dietary plants, which is the case of the nominal subspecies (Gooch et al., 2015), meaning Great Bustard's dependency on the continuation of farming practices (Delibes, Corbacho, Calvo, & Fedriani, 2012; Martín, Martínez, Bautista, & Martin, 2012). This dependency, however, in the context of climate change and land‐use alteration, raises some concerns. First, strong human disturbance exists in farmlands, where, for example, sheep compete for food and illegal hunting with dogs stresses bustards. Farmers occasionally use poisons such as carbofuran to protect the winter wheat from sheep and this has caused bustard mortality (Tian, 2003). Second, the Great Bustard appears to be using agricultural plants as a dietary source, which makes the Great Bustard sensitive to a change in planting patterns. For example, farmers tend to grow crops of high economic value such as cotton, which does not provide energy‐rich food and suitable roosting sites. The transformation from cereal crops to fruit trees has also resulted in reduced and patchy wintering habitat. Third, herbicides reduce the diversity and availability of weeds in the farmland, resulting in the decline in many species of farmland bird (Moreby & Southway, 1999). Herbicides have been applied in larger quantities in the northeast than anywhere else in China (Su, 2004), and the influence of herbicides on Asian Great Bustard needs further study. Notably, winter crop seeds are often from crop plants treated with pesticides, and these pesticide residues may cause toxic effects to Asian Great Bustard (Wang, 2012). Similarly, the use of pesticide‐coated seeds generated what was deemed an unacceptable risk on red‐legged partridge (*Alectoris rufa*) (Lopez‐Antia, Feliu, Camarero, Ortiz‐Santaliestra, & Mateo, 2016). Lastly, given the changing human landscape and dietary preference of bustards, patrolling and monitoring should be strengthened with conservation plans developed to respond to the potential climate‐related threats such as heavy snowfall events.

## CONFLICT OF INTERESTS

We have no competing interests.
