# Supplementary material for: Meta‐barcoding insights into the spatial and temporal dietary patterns of the threatened Asian Great Bustard (Otis tarda dybowskii) with potential implications for diverging migratory strategies
Source: Ecol Evol. 2018 Jan 8;8(3):1736–45. doi: 10.1002/ece3.3791 (PMC5792609; doi:10.1002/ece3.3791)
Supplement: Supplementary file 1 [file ECE3-8-1736-s001.docx]

Supplementary Fig.1 Population size changes of Asian great bustard during wintering and breeding season in Tumuji (TMJ) National Nature Reserve. For wintering season, the 1997-2004 census data are from Li et al.(2005), 2005 from Yu et al.(2009), 2006-2008 from Yi et al.(), 2009-2010 absent, 2011-2015 from the monitoring report conducted by Morigen Han and Jingying Zhou, Jie Bai and Gang Liu. For breeding season, the 1997-1999 census data are absent, 2000-2001 from Zhao et al.(?), 2002 absent, 2003 from Li et al.(2005), 2004-2006 from Zhao et al. (?), 2007-2008 from Yi et al. (), 2009-2012 absent, and 2013-2015 from the monitoring report conducted by Morigen Han and Jingying Zhou, Jie Bai and Gang Liu.


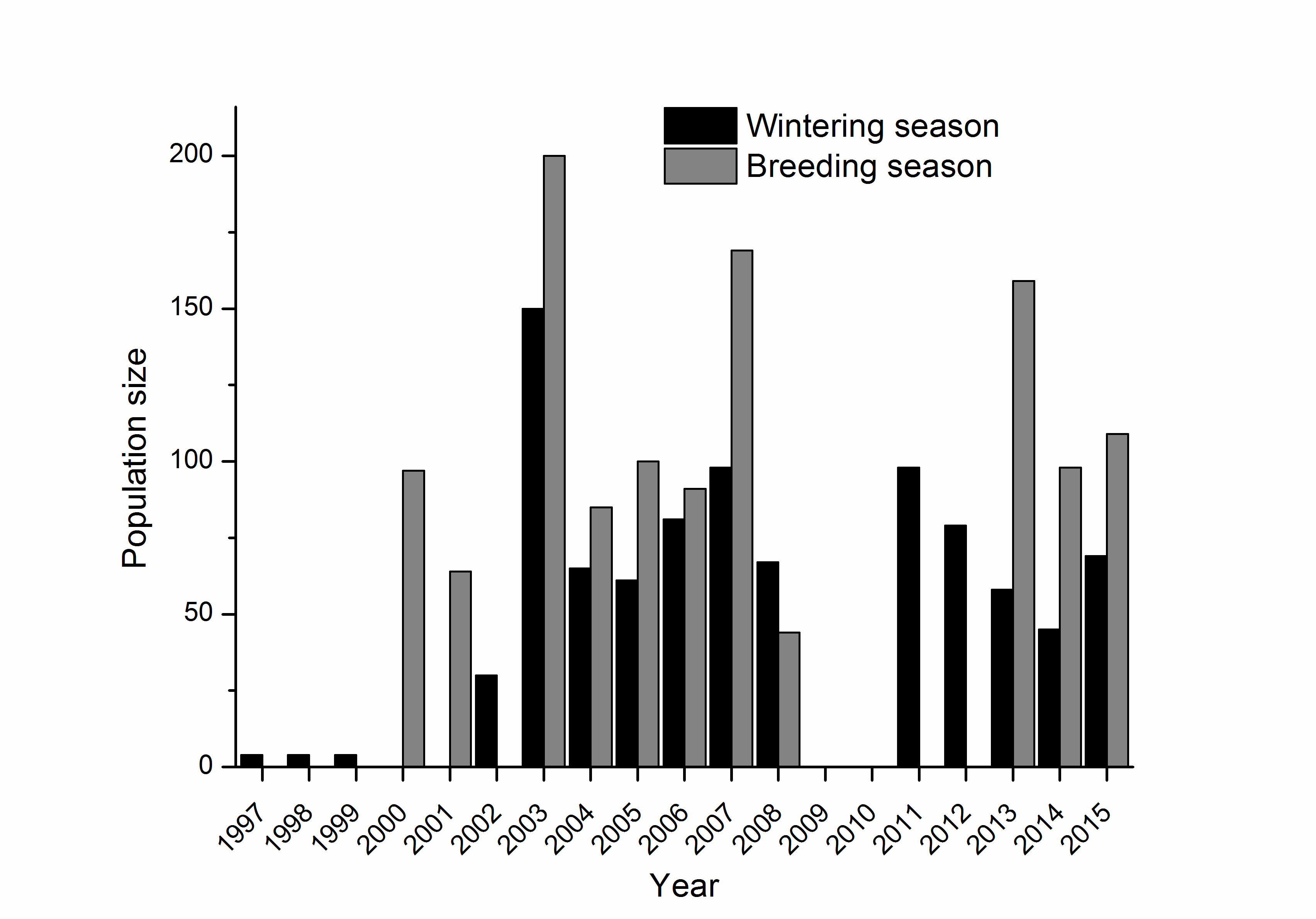


Supplementary Fig. 2 Rarefaction curve for molecular operational taxonomic units (MOTUs) at the species level in the fecal samples of Asian Great Bustard. Lines are mean estimates. TMJ, CZ and WN represent Tumuji Nature Reserve, Cangzhou and Weinan, respectively.


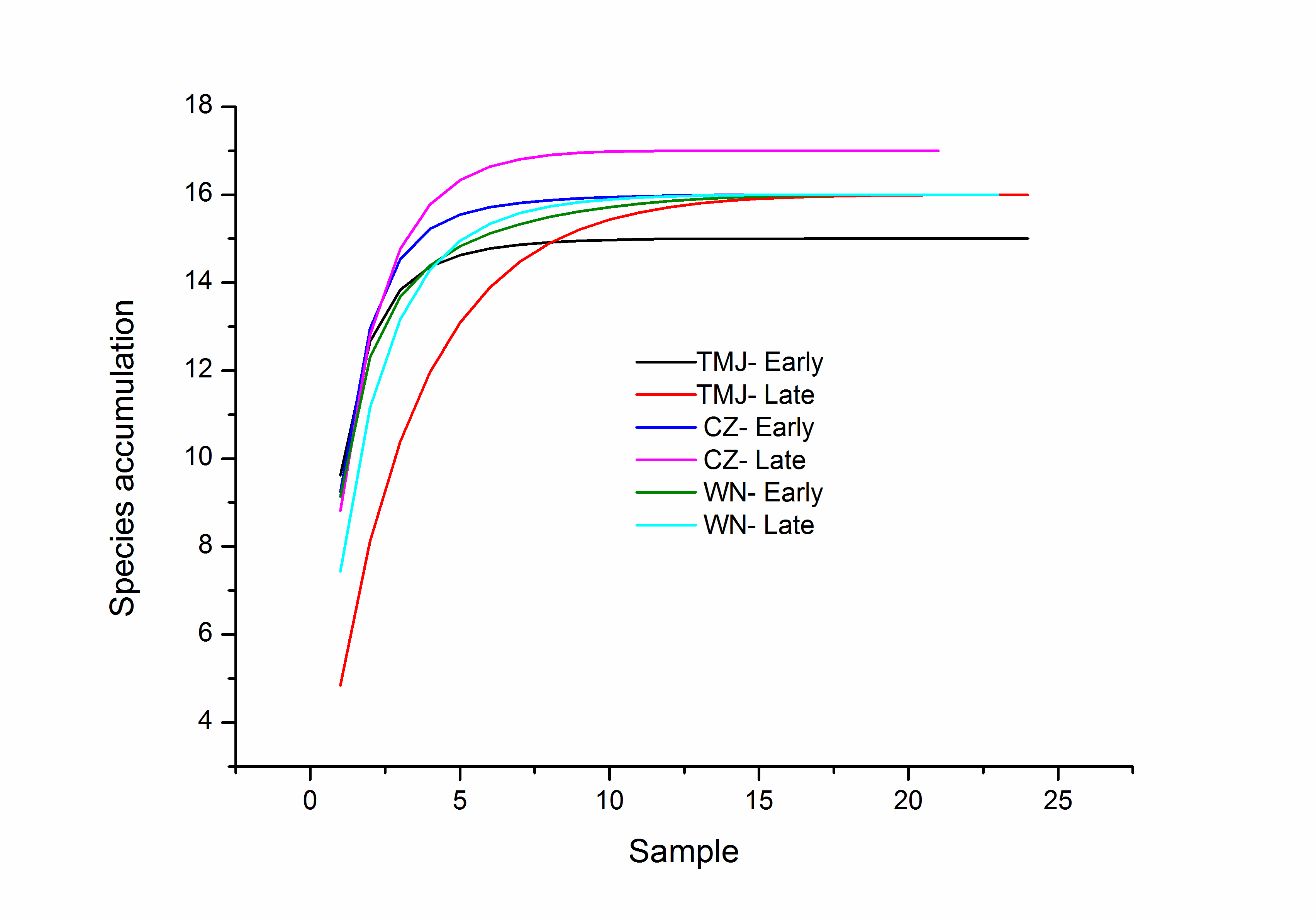


Supplementary Fig. 3 Percent occurrence of plant taxa at the family level identified in fecal samples of Asian Great Bustard from two migratory populations and one residential population during late winter and early winter. TMJ, CZ and WN represent Tumuji Nature Reserve, Cangzhou and Weinan, respectively.


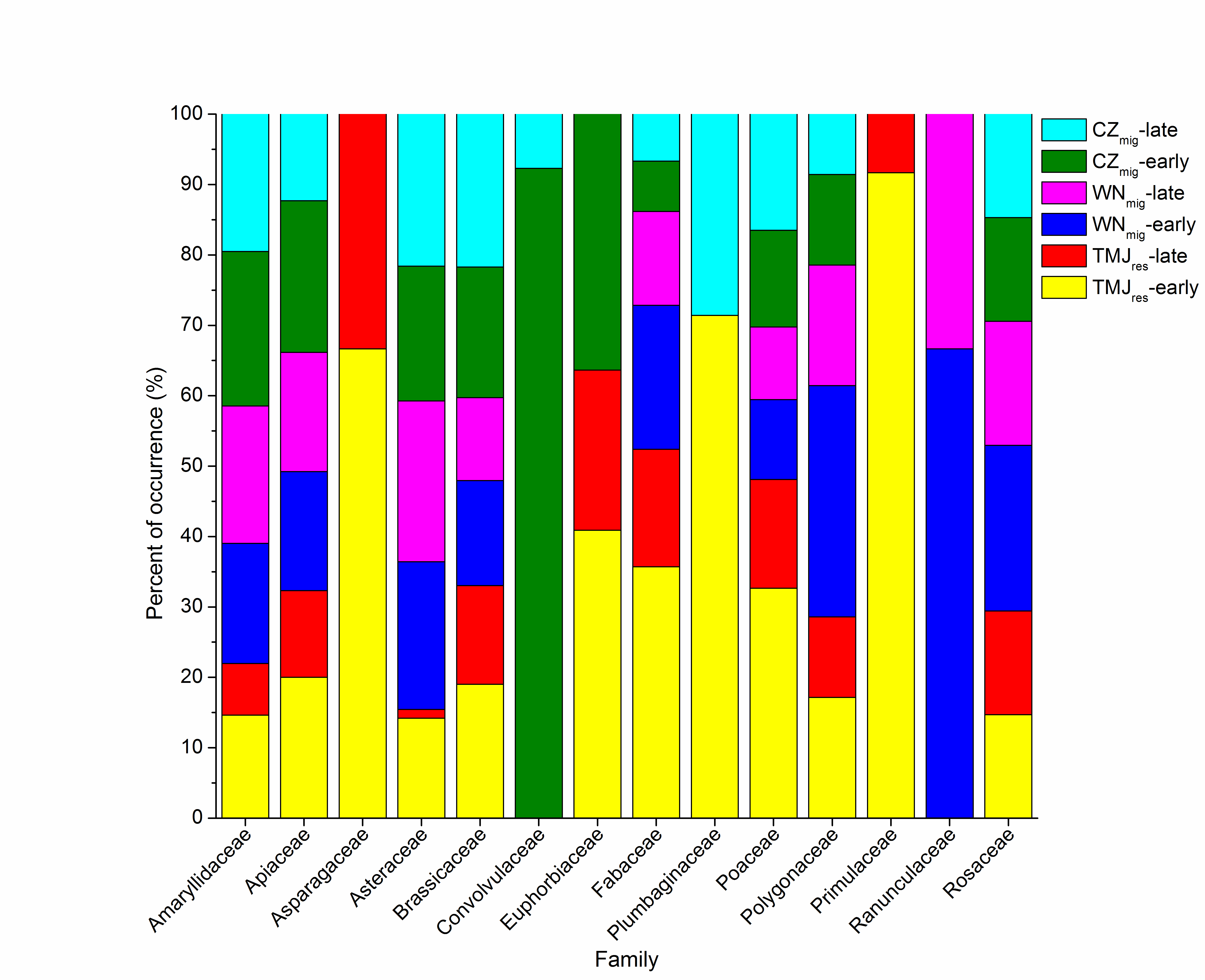


Supplementary Fig. 4 Mean fecal occurrence frequency (%) of weeds and cultivated plants in diets of Asian Great Bustard during early winter and late winter. TMJ, CZ and WN represent Tumuji Nature Reserve, Cangzhou and Weinan, respectively.

Supplementary Fig. 5 NMDS plot of dietary changes of Asian great bustard in early winter and late winter across three sites (TMJ, CZ and WN represent Tumuji Nature Reserve, Cangzhou and Weinan, respectively). (a) three sites in early winter, (b) three sites in late winter, (c) TMJ-early vs TMJ-late, (d) WN-early vs WN-late, (e) CZ-early vs CZ-late, (f) all sites in early and late winter. Aqua triangle - TMJ-early, blue inverse triangle – TMJ-late , green square – WN-winter, purple diamond – WN-late, red square – CZ-early, black triang – CZ- late.


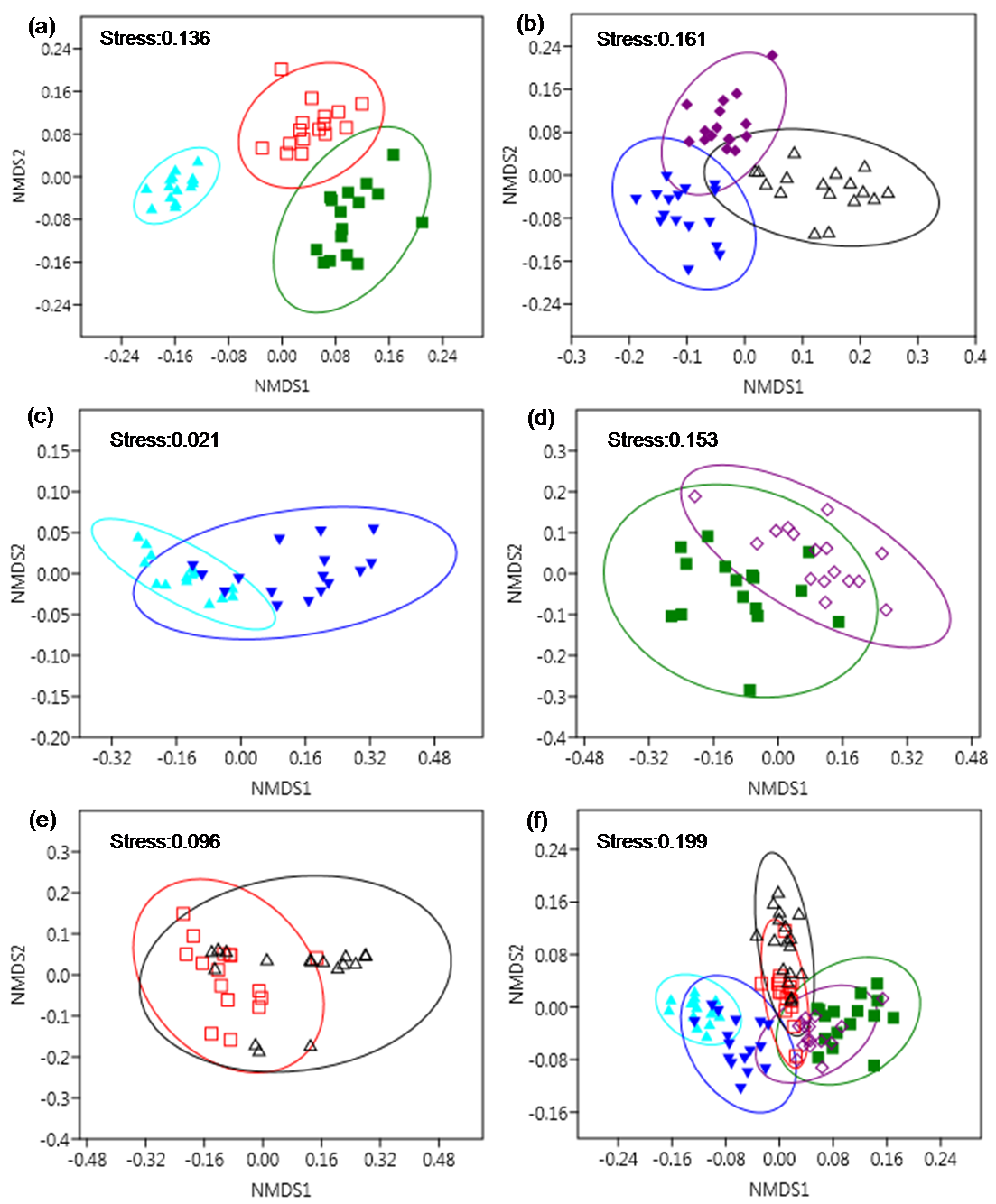


Supplementary Table 1 Primers used to amplify the universal P6 loop of the chloroplast trnL (UAA) intron, and the barcodes used (lowercase letters in red). TMJ, CZ and WN represent Tumuji Nature Reserve, Cangzhou and Weinan, respectively.

| ID | Primer | Primer sequence(5'to3') | Length | Barcodes |
| --- | --- | --- | --- | --- |
| 1 | plantG1 | ggctacGGGCAATCCTGAGCCAA | 23 | ggctac |
| 2 | plantH1 | ttctcgCCATTGAGTCTCTGCACCTATC | 28 | ttctcg |
| 3 | plantG2 | gaactaGGGCAATCCTGAGCCAA | 23 | gaacta |
| 4 | plantH2 | aagacaCCATTGAGTCTCTGCACCTATC | 28 | aagaca |
| 5 | plantG3 | ttgagtGGGCAATCCTGAGCCAA | 23 | ttgagt |
| 6 | plantH3 | aagcagCCATTGAGTCTCTGCACCTATC | 28 | aagcag |
| 7 | plantG4 | cacgtaGGGCAATCCTGAGCCAA | 23 | cacgta |
| 8 | plantH4 | taacatCCATTGAGTCTCTGCACCTATC | 28 | taacat |
| 9 | plantG5 | cactctGGGCAATCCTGAGCCAA | 23 | cactct |
| 10 | plantH5 | ggtcgaCCATTGAGTCTCTGCACCTATC | 28 | ggtcga |
| 11 | plantG6 | acaaccGGGCAATCCTGAGCCAA | 23 | acaacc |
| 12 | plantH6 | tcagagCCATTGAGTCTCTGCACCTATC | 28 | tcagag |
| 13 | plantG7 | agcactGGGCAATCCTGAGCCAA | 23 | agcact |
| 14 | plantH7 | acacaaCCATTGAGTCTCTGCACCTATC | 28 | acacaa |
| 15 | plantG8 | tcgttaGGGCAATCCTGAGCCAA | 23 | tcgtta |
| 16 | plantH8 | acaggtCCATTGAGTCTCTGCACCTATC | 28 | acaggt |
| 17 | plantG9 | gtgatcGGGCAATCCTGAGCCAA | 23 | gtgatc |
| 18 | plantH9 | tatctgCCATTGAGTCTCTGCACCTATC | 28 | tatctg |
| 19 | plantG | aacaacGGGCAATCCTGAGCCAA | 23 | aacaac |
| 20 | plantH | aaccgaCCATTGAGTCTCTGCACCTATC | 28 | aaccga |

Supplementary Table 2 Local plant reference library of specimens from study sites. TMJ, CZ and WN represent Tumuji Nature Reserve, Cangzhou and Weinan, respectively.

| Num. | Scientific name | Family | Genus | Length (bp) | Location | Accession number |
| --- | --- | --- | --- | --- | --- | --- |
| 1 | *Adenophora stricta* | Campanulaceae | Adenophora | 89 | TMJ | KY688938 |
| 2 | *Agrostis stolonifera* | Poaceae | Agrostis | 92 | TMJ | KY688939 |
| 3 | *Allium mongolicum* | Liliaceae | Allium sp. | 86 | TMJ | KY688940 |
| 4 | *Androsace umbellata* | Primulaceae | AndrosaceL | 87 | TMJ | KY178329 |
| 5 | *Anemarrhena asphodeloides* | Liliaceae | Anemarrhena | 92 | TMJ | KY178330 |
| 6 | *Artemisia capillaris* | Compositae | Artemisia | 88 | TMJ | KY688941 |
| 7 | *Arthraxon hispidus* | Gramineae | Arthraxon | 91 | TMJ | KY688942 |
| 8 | *Arundinella hirta* | Gramineae | Arundinelleae | 88 | TMJ | KY688943 |
| 9 | *Asparagus dauricus* | Liliaceae | Asparagus | 92 | TMJ | KY688944 |
| 10 | *Astragalus scaberrimus* | Leguminosae | Astragalus | 104 | TMJ | KY688945 |
| 11 | *Belamcanda chinensis* | Iridaceae | Belamcanda | 95 | TMJ | KY688946 |
| 12 | *Bolboschoenus planiculmis* | Cyperaceae | *Bolboschoenus* | 113 | TMJ | KY688947 |
| 13 | *Bupleurum chinense* | Umbelliferae | Bupleurum | 89 | TMJ | KY178336 |
| 14 | *Chloris virgata* | Gramineae | Chloris | 90 | TMJ | KY178333 |
| 15 | *Cirsium arvense* | Compositae | Cirsium | 85 | TMJ | KY688948 |
| 16 | *Clematis hexapetala* | Ranunculaceae | Clematis | 93 | TMJ | KY688949 |
| 17 | *Cymbaria daurica* | Scrophulariaceae | Cymbaria | 80 | TMJ | KY688950 |
| 18 | *Cynanchum paniculatum* | Asclepiadaceae | Cynanchum | 81 | TMJ | KY688951 |
| 19 | *Cynanchum thesioides* | Asclepiadaceae | Cynanchum | 75 | TMJ | KY688952 |
| 20 | *Cynodon dactylon* | Gramineae | Cynodon | 91 | TMJ | KY688953 |
| 21 | *Dactylorhiza hatagirea* | Orchidaceae | Dactylorhiza | 75 | TMJ | KY688954 |
| 22 | *Dysphania aristata* | Chenopodiaceae | Chenopodium | 95 | TMJ | KY688955 |
| 23 | *Eleocharis uniglumis* | Cyperaceae | Heleocharis | 160 | TMJ | KY688956 |
| 24 | *Festuca ovina* | Gramineae | Festuca | 92 | TMJ | KY688957 |
| 25 | *Filifolium sibiricum* | Compositae | Filifolium | 90 | TMJ | KY688958 |
| 26 | *Fimbristylis dichotoma* | Cyperaceae | Fimbristylis | 113 | TMJ | KY688959 |
| 27 | *Galium verum* | Rubiaceae | Galium | 71 | TMJ | KY688960 |
| 28 | *Glaux maritima* | Primulaceae | Glaux | 84 | TMJ | KY688961 |
| 29 | *Glycine max* | Leguminosae | Glycine | 90 | WN | KY688962 |
| 30 | *Glycyrrhiza uralensis* | Leguminosae | Glycyrrhiza | 90 | TMJ | KY688963 |
| 31 | *Gypsophila davurica* | Caryophyllaceae | Gypsophila | 101 | TMJ | KY688964 |
| 32 | *Halerpestes cymbalaria* | Ranunculaceae | Halerpestes | 89 | TMJ | KY688965 |
| 33 | *Haplophyllum dauricum* | Rutaceae | Haplophyllum | 85 | TMJ | KY688966 |
| 34 | *Hordeum roshevitzii* | Gramineae | Hordeum | 86 | TMJ | KY688967 |
| 35 | *Iris bungei* | Iridaceae | Iris | 89 | TMJ | KY688968 |
| 36 | *Iris lactea* | Iridaceae | Iris | 89 | TMJ | KY688969 |
| 37 | *Iris dichotoma* | Iridaceae | Iris | 95 | TMJ | KY688970 |
| 38 | *Juncus effusus* | Juncaceae | Juncus | 89 | TMJ | KY688971 |
| 39 | *Leibnitzia anandria* | Compositae | Gerbera | 89 | TMJ | KY688972 |
| 40 | *Leontopodium leontopodioides* | Compositae | Leontopodium | 91 | TMJ | KY688973 |
| 41 | *Lespedeza davurica* | Leguminosae | Lespedeza | 90 | TMJ | KY688974 |
| 42 | *Leymus chinensis* | Gramineae | Leymus | 91 | TMJ | KY178332 |
| 43 | *Lysimachia fortunei* | Primulaceae | Lysimachia | 84 | TMJ | KY688975 |
| 44 | *Mazus stachydifolius* | Scrophulariaceae | Mazus | 89 | TMJ | KY688976 |
| 45 | *Medicago sativa* | Leguminosae | Medicago | 94 | TMJ | KY688977 |
| 46 | *Oxytropis racemosa* | Leguminosae | Oxytropis | 99 | TMJ | KY688978 |
| 47 | *Parthenocissus tricuspidata* | Euphorbiaceae | Euphorbia | 94 | TMJ | KY688979 |
| 48 | *Phragmites australis* | Gramineae | Phragmites | 91 | CZ | KY688980 |
| 49 | *Plantago asiatica* | Plantaginaceae | Plantago | 80 | TMJ | KY688981 |
| 50 | *Polygala sibirica* | Polygalaceae | Polygala | 92 | TMJ | KY688982 |
| 51 | *Senecio dubitabilis* | Compositae | Senecio | 93 | TMJ | KY688983 |
| 52 | *Polygonum sibiricum* | Polygonaceae | Polygonum | 71 | TMJ | KY688984 |
| 53 | *Potentilla anserina* | Rosaceae | Potentilla | 87 | TMJ | KY688985 |
| 54 | *Potentilla discolor* | Rosaceae | Potentilla | 91 | WN | KY178331 |
| 55 | *Potentilla flagellaris* | Rosaceae | Potentilla | 78 | TMJ | KY688986 |
| 56 | *Potentilla fragarioides* | Rosaceae | Potentilla | 91 | TMJ | KY688987 |
| 57 | *Primula farinosa* | Primulaceae | Primula | 77 | TMJ | KY688988 |
| 58 | *Prunus sibirica* | Rosaceae | Armeniaca | 90 | TMJ | KY688989 |
| 59 | *Puccinellia distans* | Gramineae | Puccinellia | 92 | TMJ | KY688990 |
| 60 | *Poa annua* | Gramineae | Poa | 97 | TMJ | KY688991 |
| 61 | *Rhaponticum uniflorum* | Compositae | *Rhaponticum* | 89 | TMJ | KY178334 |
| 62 | *Sanguisorba officinalis* | Rosaceae | Sanguisorba | 91 | TMJ | KY688992 |
| 63 | *Scabiosa comosa* | Dipsacaceae | Scabiosa | 88 | TMJ | KY178335 |
| 64 | *Scutellaria baicalensis* | Labiatae | Scutellaria | 89 | TMJ | KY688993 |
| 65 | *Setaria pumila* | Gramineae | Setaria | 91 | WN | KY688994 |
| 66 | *Solanum rostratum* | Solanaceae | Solanum | 79 | TMJ | KY688995 |
| 67 | *Spodiopogon sibiricus* | Gramineae | Spodiopogon | 91 | TMJ | KY688996 |
| 68 | *Stellera chamaejasme* | Euphorbiaceae | Euphorbia | 100 | TMJ | KY688997 |
| 69 | *Stipa baicalensis* | Gramineae | Stipa | 78 | TMJ | KY688998 |
| 70 | *Thalictrum petaloideum* | Ranunculaceae | Thalictrum | 93 | TMJ | KY688999 |
| 71 | *Thesium chinense* | Santalaceae | Thesium | 141 | TMJ | KY689000 |
| 72 | *Triglochin palustris* | Potamogetonaceae | Triglochin | 143 | TMJ | KY689001 |
| 73 | *Turczaninovia fastigiata* | Compositae | Turczaninowia | 89 | TMJ | KY689002 |
| 74 | *Vigna radiata* | Leguminosae | Vigna | 90 | TMJ | KY178328 |
| 75 | *Xanthium strumarium* | Compositae | Xanthium | 89 | TMJ | KY689003 |
| 76 | *Zea mays* | Gramineae | Zea | 90 | TMJ | KY178327 |
| 77 | *Triticum aestivum* | Gramineae | Triticum | 90 | CZ | KY178322 |
| 78 | *Potentilla ancistrifolia* | Rosaceae | Potentilla | 91 | WN | KY178336 |

Supplementary Table 3 List of corresponding plant taxa identified in fecal samples of Asian Great Bustard three populations in early winter and late winter. Taxonomic assignations identified by local reference library are highlighted in bold. TMJ, WN and CZ represent Tumuji Nature Reserve, Weinan and Cangzhou, respectively.

| Sequence ID | Family | Species | Number of sequences reads (Frequency of occurrence: number of samples) | | | | | |
| --- | --- | --- | --- | --- | --- | --- | --- | --- |
|  |  |  | TMJ-early (15) | TMJ-late (16) | WN-early (16) | WN-late (16) | CZ-early (16) | CZ-late (17) |
| GB01 | Amaryllidaceae | *Allium mongolicum* | 801 (6) | 355 (3) | 0 | 0 | 0 | 0 |
| GB02 |  | *Allium macrostemon* | 0 () | 0 | 1078 (7) | 832 (8) | 1670 (16) | 1023 (17) |
| GB03 | Apiaceae | *Unknown* | 2631 (13) | 638 (8) | 4750 (11) | 1257 (11) | 3686 (9) | 2753 (8) |
| GB04 | Asparagaceae | *Unknown* | 940 (2) | 270 (1) | 0 | 0 | 0 | 0 |
| GB05 | Asteraceae | *Artemisia spp.* | 2502 (12) | 21 (1) | 0 | 0 | 0 | 0 |
| GB06 |  | *Unknown* | 973 (11) | 36 (1) | 0 | 0 | 0 | 0 |
| GB07 |  | *Adenocaulon bicolor* | 0 | 0 | 6458 (11) | 40790 (11) | 0 | 0 |
| GB08 |  | *Unknown* | 0 | 0 | 1123 (11) | 11453 (10) | 0 | 0 |
| GB09 |  | *Unknown* | 0 | 0 | 4852 (12) | 3828 (13) | 0 | 0 |
| GB10 |  | *Unknown* | 0 | 0 | 0 | 2733 (3) | 0 | 0 |
| GB11 |  | *Unknown* | 0 | 0 | 0 | 0 | 17507 (14) | 3155 (8) |
| GB12 |  | *Unknown* | 0 | 0 | 0 | 0 | 47 (1) | 19510 (13) |
| GB13 |  | *Unknown* | 0 | 0 | 0 | 0 | 5952 (15) | 2943 (11) |
| GB14 |  | *Unknown* | 0 | 0 | 0 | 0 | 92 (1) | 2598 (10) |
| GB15 | Brassicaceae | *Descurainia sophia* | 21807 (15) | 8268 (16) | 5777 (15) | 5152 (15) | 73403 (14) | 744732 (16) |
| GB16 |  | *Unknown* | 856 (9) | 1768 (9) | 548 (12) | 700 (3) | 4142 (13) | 1261 (1) |
| GB17 |  | *Unknown* | 978 (6) | 210 (2) | 0 | 0 | 0 | 0 |
| GB18 |  | *Unknown* | 983 (12) | 114 (4) | 0 | 0 | 0 | 0 |
| GB19 |  | *Rorippa spp.* | 0 | 0 | 3254 (6) | 2141 (8) | 0 | 0 |
| GB20 |  | *Unknown* | 0 | 0 | 0 | 0 | 1353 (11) | 8078 (7) |
| GB21 |  | *Ricotia spp.* | 0 | 0 | 0 | 0 | 77 (1) | 1462 (8) |
| GB22 | Convolvulaceae |  | 0 | 0 | 0 | 0 | 2967 (12) | 39 (1) |
| GB23 | Euphorbiaceae | *Euphorbia esula* | 1049 (7) | 0 | 0 | 0 | 0 | 0 |
| GB24 |  | *Acalypha spp.* | 240 (2) | 800 (5) | 0 | 0 | 0 | 0 |
| GB25 |  | *Acalypha australis* | 0 | 0 | 0 | 0 | 3445 (8) | 0 |
| GB26 | Fabaceae | *Vigna radiata* | 1806061 (15) | 300691 (16) | 0 | 0 | 0 | 0 |
| GB27 |  | *Oxytropis racemosa* | 35282 (15) | 672 (7) | 0 | 0 | 0 | 0 |
| GB28 |  | *Fabaceae1* | 5704 (15) | 804 (6) | 1101 (16) | 66 (1) | 0 | 0 |
| GB29 |  | *Fabaceae2* | 1780 (12) | 63 (1) | 0 | 0 | 0 | 0 |
| GB30 |  | *Fabaceae3* | 955 (6) | 120 (1) | 0 | 0 | 0 | 0 |
| GB31 |  | *Medicago sativa* | 854 (9) | 212 (2) | 478 (11) | 1960 (8) | 0 | 0 |
| GB32 |  | *Fabaceae4* | 790 (3) | 275 (2) | 0 | 0 | 0 | 0 |
| GB33 |  | *Glycine max* | 0 | 0 | 284333 (6) | 13305 (16) | 3768 (15) | 2564 (14) |
| GB34 |  | *Fabaceae5* | 0 | 0 | 3686 (10) | 213 (2) | 0 | 0 |
| GB35 | Plumbaginaceae | *Limonium spp.1* | 1428 (5) | 0 | 0 | 0 | 0 | 0 |
| GB36 |  | *Limonium spp.2* | 0 | 0 | 0 | 0 | 0 | 5816 (2) |
| GB37 | Poaceae | *Zea mays* | 190487 (15) | 15533 (16) | 3068 (1) | 3071 (8) | 26875 () | 5155 (16) |
| GB38 |  | *Leymus chinensis* | 4613 (14) | 1483 (13) | 0 | 0 | 0 | 83 (1) |
| GB39 |  | *Chloris virgata* | 462 (2) | 4955 (8) | 0 | 0 | 0 | 131 (2) |
| GB40 |  | *Puccinellia distans* | 1384 (9) | 45 (1) | 0 | 0 | 25 (1) | 30318 (13) |
| GB41 |  | *Poaceae1* | 1384 (12) | 37 (1) | 0 | 0 | 0 | 0 |
| GB42 |  | *Phragmites communis* | 1066 (10) | 0 | 0 | 0 | 0 | 0 |
| GB43 |  | *Calamagrostis spp.1* | 1049 (11) | 0 | 0 | 0 | 0 | 0 |
| GB44 |  | *Poaceae2* | 1042 (10) | 0 | 0 | 0 | 0 | 0 |
| GB45 |  | *Poaceae3* | 902 (10) | 122 (1) | 0 | 0 | 0 | 0 |
| GB46 |  | *Avena spp.* | 335 (2) | 669 (5) | 94 (10) | 7015 (8) |  | 0 |
| GB47 |  | *Triticum aestivu* | 0 | 0 | 5194 (1) | 1426 (15) | 23062 (16) | 53077 (16) |
| GB48 |  | *Hordeum spp.* | 0 | 0 | 4639 (10) | 37 (1) | 0 | 0 |
| GB49 |  | *Calamagrostis spp.2* | 0 | 0 | 993 (14) | 111 (3) | 0 | 0 |
| GB50 |  | *Setaria glauca* | 0 | 0 | 0 | 0 | 1868 (8) | 77 (1) |
| GB51 |  | *Phragmites australis* | 0 | 0 | 0 |  | 0 | 1274 (6) |
| GB52 | Polygonaceae | *Rumex spp.1* | 5554 (12) | 1904 (9) | 83982 (5) | 1947 (12) | 2025 (9) | 1296 (2) |
| GB53 |  | *Rumex spp.2* |  |  | 1592 (3) | 0 | 0 | 0 |
| GB54 | Primulaceae | *Androsace spp.* | 3512 (11) | 116 (1) | 0 | 0 | 0 | 0 |
| GB55 | Ranunculaceae | *Ranunculus sceleratus* | 0 | 0 | 131 (2) | 899 (1) | 0 | 0 |
| GB56 | Rosaceae | *Potentilla discolor* | 26431 (15) | 10671 (15) | 152114 (16) | 94244 (16) | 7833 (15) | 8476 (15) |
| GB57 |  | *Potentilla ancistrifolia* | 0 | 0 | 1202 (7) | 435 (2) | 0 | 0 |

Supplementary Table 4 Differences in Simpson diversity index between wintering sites and between wintering time. Simpson diversity index results comparing diet pairwise and overall sampling sites. TMJ, CZ and WN represent Tumuji Nature Reserve, Cangzhou and Weinan, respectively.

| Test | Wintering site | Wintering time | Normality Test | Statistical result |
| --- | --- | --- | --- | --- |
| Simpson diversity index |  |  |  |  |
| Between wintering time | TMJ | Early × Late | P = 0.20 | t_29_ = -1.33, P <0.01 |
|  | WN | Early × Late | P = 0.02 | Z = -1.55, P = 0.12 |
|  | CZ | Early × Late | P <0.01 | Z = -3.57, P =0.02 |
| Between wintering site | TMJ×WN×CZ | Early winter | P = 0.055 | F_2,44_ = 13.85, P <0.01 |
|  | TMJ×WN | Early winter |  | P = 0.02 |
|  | TMJ×CZ | Early winter |  | P <0.01 |
|  | WN×CZ | Early winter |  | P = 0.06 |
|  | TMJ×WN×CZ | Late winter | P = 0.17 | F_2,46_ = 11.96, P <0.01 |
|  | TMJ×WN | Late winter |  | P <0.01 |
|  | TMJ×CZ | Late winter |  | P = 0.72 |
|  | WN×CZ | Late winter |  | P <0.01 |
| Chao1 diversity index |  |  |  |  |
| Between wintering time | TMJ | Early × Late | P = 0.02 | Z = -4.02, P <0.01 |
|  | WN | Early × Late | P = 0.57 | t_30_ = 1.45, P =0.15 |
|  | CZ | Early × Late | P =0.27 | t_31_ = 1.07, P =0.29 |
| Between wintering site | TMJ×WN×CZ | Early winter | P = 0.28 | F_2,44_ = 12.55, P <0.01 |
|  | TMJ×WN | Early winter |  | P <0.01 |
|  | TMJ×CZ | Early winter |  | P <0.01 |
|  | WN×CZ | Early winter |  | P = 0.84 |
|  | TMJ×WN×CZ | Late winter | P = 0.31 | F_2,46_ = 0.50, P =0.61 |
|  | TMJ×WN | Late winter |  | P =0.71 |
|  | TMJ×CZ | Late winter |  | P = 0.61 |
|  | WN×CZ | Late winter |  | P =0.98 |

Supplementary Table 5 Beta diversity results for interactions between wintering site and wintering time using Adonis function.

|  | Df | Sums Of Sqs | Mean Sqs | F. Model | R^2^ | P |
| --- | --- | --- | --- | --- | --- | --- |
| Site | 2 | 11.492 | 5.7460 | 26.4910 | 0.31276 | <0.001 |
| Time | 1 | 1.716 | 1.7165 | 7.9135 | 0.04671 | <0.001 |
| Site:Time | 2 | 4.015 | 2.0073 | 9.2543 | 0.10926 | <0.001 |
| Residuals | 90 | 19.521 | 0.2169 |  | 0.53127 |  |
| Total | 95 | 36.744 |  |  | 1.00000 |  |

Supplementary Table 6 Results of beta diversity analysis using the the R Vegan package – function Adonis and Betadisper. TMJ, CZ and WN represent Tumuji Nature Reserve, Cangzhou and Weinan, respectively.

| Test | Wintering sites | Wintering time | Statistical result |
| --- | --- | --- | --- |
| Between wintering period | TMJ | Early × Late | F_1,29_=18.32, P < 0.001 |
|  | WN | Early × Late | F_1,30_=6.90, P < 0.001 |
|  | CZ | Early × Late | F_1,31_ = 6.90, P = 0.001 |
| Between wintering area | TMJ×WN×CZ | Early | F_2,44_=7.98, P=0.001 |
|  | TMJ×WN | Early | F_1,29_=27.46, P < 0.001 |
|  | TMJ×CZ | Early | F_1,29_=28.44, P < 0.001 |
|  | WN×CZ | Early | F_1,30_=6.90, P < 0.001 |
|  | TMJ×WN×CZ | Late | F_2,46_=14.87, P < 0.001 |
|  | TMJ×WN | Late | F_1,30_=16.30, P < 0.001 |
|  | TMJ×CZ | Late | F_1,31_=16.549, P < 0.001 |
|  | WN×CZ | Late | F_1,31_=12.192, P < 0.001 |
| Considering interactive effects | ALL | Both | F_2,90_=9.25, R^2^=0.11, P<0.001 |

Supplementary Table 7 Differences in diet composition between wintering sites and between wintering time. ANOSIM results comparing diet pairwise and over all sampling sites. TMJ, CZ and WN represent Tumuji Nature Reserve, Cangzhou and Weinan, respectively.

| Test | Wintering sites | Wintering time | Statistical result |
| --- | --- | --- | --- |
| Between wintering period | TMJ | Early × Late | R=0.56, P=0.001 |
|  | WN | Early × Late | R=0.41, P=0.001 |
|  | CZ | Early × Late | R=0.28，P=0.003 |
| Between wintering area | TMJ×WN×CZ | Early | R=0.93, P=0.001 |
|  | TMJ×WN×CZ | Late | R=0.81, P=0.001 |
|  | TMJ×WN | Early | R=0.99, P=0.001 |
|  | TMJ×CZ | Early | R=0.98, P=0.001 |
|  | WN×CZ | Early | R=0.81, P=0.001 |
|  | TMJ×WN | Late | R=0.84, P=0.001 |
|  | TMJ×CZ | Late | R=0.90, P=0.001 |
|  | WN×CZ | Late | R=0.76, P=0.001 |
| All sampling sites | TMJ×WN×CZ | Both | R=0.75, P=0.001 |
| All sampling time | All | Early × Late | R=0.07, P=0.091 |

Supplementary Table 8 Results of the similarity percentage (SIMPER) analysis among the three wintering sites during early winter and late winter. TMJ, CZ and WN represent Tumuji Nature Reserve, Cangzhou and Weinan, respectively.

| **In early winter** | | | | | | |
| --- | --- | --- | --- | --- | --- | --- |
| **TMJ versus CZ Average dissimilarity= 95.32** | | | | | | |
| **Species** | **Average abundance** | | **Average dissimilarity** | **Dissimilarity**  **/SD** | **Contribution%** | **Cumulative%** |
|  | **TMJ** | **CZ** |  |  |  |  |
| *Vigna radiata* | 120404.07 | 0.00 | 77.10 | 8.44 | 80.88 | 80.88 |
| *Zea mays* | 12699.13 | 12699.13 | 7.21 | 1.53 | 7.57 | 88.45 |
| *Descurainia sophia* | 1453.80 | 4587.69 | 3.08 | 0.48 | 3.23 | 91.68 |
| **TMJ versus WN Average dissimilarity= 97.68** | | | | | | |
| **Species** | **Average abundance** | | **Average dissimilarity** | **Dissimilarity**  **/SD** | **Contribution%** | **Cumulative%** |
|  | **TMJ** | **WN** |  |  |  |  |
| *Vigna radiata* | 120404.07 | 0.00 | 66.50 | 4.26 | 68.08 | 68.08 |
| *Glycine max* | 0.00 | 17770.81 | 11.25 | 0.89 | 11.52 | 79.59 |
| *Zea mays* | 12699.13 | 191.75 | 6.73 | 1.57 | 6.89 | 86.48 |
| *Potentilla discolor* | 1762.07 | 9507.13 | 5.34 | 0.63 | 5.46 | 91.95 |
| **WN versus CZ Average dissimilarity= 91.44** | | | | | | |
| **Species** | **Average abundance** | | **Average dissimilarity** | **Dissimilarity**  **/SD** | **Contribution%** | **Cumulative%** |
|  | **WN** | **CZ** |  |  |  |  |
| *Glycine max* | 17770.81 | 235.50 | 34.91 | 1.39 | 38.18 | 38.18 |
| *Potentilla discolor* | 9507.13 | 489.56 | 15.66 | 0.85 | 17.13 | 55.31 |
| *Descurainia sophia* | 361.06 | 4587.69 | 11.73 | 0.75 | 12.83 | 68.13 |
| Rumex spp.1 | 5248.88 | 126.56 | 9.28 | 0.52 | 10.15 | 78.28 |
| *Zea mays* | 191.75 | 1679.69 | 4.19 | 0.59 | 4.58 | 82.86 |
| *Triticum aestivum* | 324.63 | 1441.38 | 4.11 | 0.45 | 4.49 | 87.36 |
| Asteraceae4 | 0.00 | 1094.19 | 2.95 | 0.59 | 3.23 | 90.59 |
| **In late winter** | | | | | | |
| **TMJ versus CZ Average dissimilarity= 96.04** | | | | | | |
| **Species** | **Average abundance** | | **Average dissimilarity** | **Dissimilarity**  **/SD** | **Contribution%** | **Cumulative%** |
|  | **TMJ** | **CZ** |  |  |  |  |
| *Descurainia sophia* | 516.75 | 43807.76 | 47.87 | 1.39 | 49.84 | 49.84 |
| *Vigna radiata* | 18793.19 | 0.00 | 29.35 | 1.16 | 30.56 | 80.40 |
| *Triticum aestivum* | 0.00 | 3122.18 | 7.17 | 0.40 | 7.47 | 87.87 |
| *Puccinellia distans* | 2.81 | 1783.41 | 2.10 | 0.30 | 2.18 | 90.05 |
| **TMJ versus WN Average dissimilarity= 91.42** | | | | | | |
| **Species** | **Average abundance** | | **Average dissimilarity** | **Dissimilarity**  **/SD** | **Contribution%** | **Cumulative%** |
|  | **TMJ** | **WN** |  |  |  |  |
| *Vigna radiata* | 18793.19 | 0.00 | 51.15 | 2.14 | 55.95 | 55.95 |
| *Potentilla discolor* | 666.94 | 5890.25 | 17.95 | 0.98 | 19.64 | 75.59 |
| *Glycine max* | 0.00 | 831.56 | 3.70 | 0.88 | 4.05 | 79.64 |
| *Adenocaulon bicolor* | 0.00 | 2549.38 | 2.58 | 0.32 | 2.82 | 82.46 |
| *Descurainia sophia* | 516.75 | 322.00 | 2.27 | 0.98 | 2.48 | 84.94 |
| *Zea mays* | 970.81 | 191.94 | 2.12 | 1.28 | 2.32 | 87.26 |
| Avena spp. | 41.81 | 438.44 | 2.06 | 0.25 | 2.26 | 89.52 |
| Asteraceae2 | 0.00 | 239.25 | 1.45 | 0.30 | 1.58 | 91.10 |
| **WN versus CZ Average dissimilarity= 93.11** | | | | | | |
| **Species** | **Average abundance** | | **Average dissimilarity** | **Dissimilarity**  **/SD** | **Contribution%** | **Cumulative%** |
|  | **WN** | **CZ** |  |  |  |  |
| *Descurainia sophia* | 322.00 | 43807.76 | 54.70 | 1.57 | 58.75 | 58.75 |
| *Potentilla discolor* | 5890.25 | 498.59 | 12.19 | 0.76 | 13.09 | 71.84 |
| *Triticum aestivum* | 89.13 | 3122.18 | 8.45 | 0.42 | 9.08 | 80.92 |
| *Puccinellia distans* | 0.00 | 1783.41 | 2.33 | 0.31 | 2.50 | 83.43 |
| *Glycine max* | 831.56 | 150.82 | 2.10 | 0.62 | 2.25 | 85.68 |
| *Adenocaulon bicolor* | 2549.38 | 0.00 | 2.06 | 0.30 | 2.22 | 87.90 |
| Asteraceae5 | 0.00 | 1147.65 | 1.56 | 0.28 | 1.68 | 89.58 |
| Avena spp. | 438.44 | 0.00 | 1.27 | 0.20 | 1.36 | 90.94 |
